# NeuroAiD^TM^-II (MLC901) Promoted Neurogenesis by Activating the PI3K/AKT/GSK-3β Signaling Pathway in Rat Spinal Cord Injury Models

**DOI:** 10.3390/biomedicines12081920

**Published:** 2024-08-21

**Authors:** Anam Anjum, Muhammad Dain Yazid, Muhammad Fauzi Daud, Jalilah Idris, Angela Min Hwei Ng, Amaramalar Selvi Naicker, Ohnmar Htwe Rashidah Ismail, Ramesh Kumar Athi Kumar, Yogeswaran Lokanathan

**Affiliations:** 1 Department of Tissue Engineering and Regenerative Medicine, Faculty of Medicine, University Kebangsaan Malaysia, Kuala Lumpur 56000, Malaysia; aanjum@som.umaryland.edu (A.A.); dain@ppukm.ukm.edu.my (M.D.Y.); angela@ppukm.ukm.edu.my (A.M.H.N.); 2 Department of Neurosurgery, University of Maryland School of Medicine, Baltimore, MD 21201, USA; 3 Institute of Medical Science Technology, Universiti Kuala Lumpur Malaysia, Kajang 43000, Malaysia; mfauzi@unikl.edu.my (M.F.D.); jalilahidris@unikl.edu.my (J.I.); 4 Department of Orthopaedics & Traumatology, Faculty of Medicine, Universiti Kebangsaan Malaysia, Kuala Lumpur 56000, Malaysia; amara@ppukm.ukm.edu.my; 5 Department of Orthopaedics and Traumatology, Faculty of Medicine, Universiti Sultan Zainal Abidin (UniSZA), Kuala Terengganu 21300, Malaysia; ohmar@ppukm.ukm.edu.my; 6 Department of Surgery, Hospital Canselor Tuanku Muhriz, Universiti Kebangsaan Malaysia, Kuala Lumpur 56000, Malaysia; rameshkumar@ppukm.ukm.edu.my; 7 Advance Bioactive Materials-Cells UKM Research Group, Universiti Kebangsaan Malaysia, Bangi 43600, Malaysia

**Keywords:** calibrated forceps compression injury, rat mechanical spinal injury model, MLC901 (NeuroAiD^TM^-II), GAP-43, GFAP, signaling pathway

## Abstract

Traumatic damage to the spinal cord (SCI) frequently leads to irreversible neurological deficits, which may be related to apoptotic neurodegeneration in nerve tissue. The MLC901 treatment possesses neuroprotective and neuroregenerative activity. This study aimed to explore the regenerative potential of MLC901 and the molecular mechanisms promoting neurogenesis and functional recovery after SCI in rats. A calibrated forceps compression injury for 15 s was used to induce SCI in rats, followed by an examination of the impacts of MLC901 on functional recovery. The Basso, Beattie, and Bresnahan (BBB) scores were utilized to assess neuronal functional recovery; H&E and immunohistochemistry (IHC) staining were also used to observe pathological changes in the lesion area. Somatosensory Evoked Potentials (SEPs) were measured using the Nicolet^®^ Viking Quest™ apparatus. Additionally, we employed the Western blot assay to identify PI3K/AKT/GSK-3β pathway-related proteins and to assess the levels of GAP-43 and GFAP through immunohistochemistry staining. The study findings revealed that MLC901 improved hind-limb motor function recovery, alleviating the pathological damage induced by SCI. Moreover, MLC901 significantly enhanced locomotor activity, SEPs waveform, latency, amplitude, and nerve conduction velocity. The treatment also promoted GAP-43 expression and reduced reactive astrocytes (GFAP). MLC901 treatment activated p-AKT reduced p-GSK-3β expression levels and showed a normalized ratio (fold changes) relative to β-tubulin. Specifically, p-AKT exhibited a 4-fold increase, while p-GSK-3β showed a 2-fold decrease in T rats compared to UT rats. In conclusion, these results suggest that the treatment mitigates pathological tissue damage and effectively improves neural functional recovery following SCI, primarily by alleviating apoptosis and promoting neurogenesis. The underlying molecular mechanism of this treatment mainly involves the activation of the PI3K/AKT/GSK-3β pathway.

## 1. Introduction

Spinal cord injury (SCI) is a serious neurological condition that can result from trauma, inflammation, tumors, or other insults. It significantly worsens patients’ quality of life and increases their emotional and economic burden [1,2]. SCI often leads to irreversible locomotor, sensory, and autonomic dysfunction below the level of the injury [2], with approximately 250,000–500,000 new cases each year [3]. Despite therapeutic advances in recent decades, effective treatments for SCI are still lacking [2,4]. While available treatments can yield favorable outcomes, they are often accompanied by side effects. As a result, traditional medicine for SCI is gaining increasing popularity [5]. 

The pathophysiological changes following spinal cord injury (SCI) occur in two stages: the primary injury phase and the secondary injury phase, each characterized by complex pathological events [2]. Immediately after SCI trauma, primary mechanical damage disrupts the blood–spinal cord barrier (BSCB), leading to further adverse changes and secondary injuries in the spinal cord [6]. Mechanical injury to the spinal cord triggers oxidative stress, disrupting the normal redox balance and leading to the generation of reactive oxygen species (ROS) [7]. Similarly, compression injuries, which are the second most common cause of mechanical injury, simulate persistent spinal canal occlusion. This condition increases the risk of oxidative damage and calcium overload, which can damage the mitochondria [8,9].

MLC901 (NeuroAiD^TM^-II) is a traditional Chinese medicine approved by the China Food and Drug Administration (CFDA) for the treatment of traumatic brain injuries [10,11,12]. MLC901 is composed of nine herbal components, including *Radix astragali*, *Radix salviae miltiorrhizae*, *Radix paeoniae rubra*, *Rhizoma chuanxiong*, *Radix angelicae sinensis*, *Carthamus tinctorius*, *Prunus persica*, *Radix polygalae*, and *Rhizoma acori tatarinowii* [12,13,14]. Numerous studies have demonstrated that MLC901 exhibits neuroprotective effects on neurons affected by ischemic damage, thereby enhancing cognitive function during post-stroke recovery [12,14,15]. The neuroregenerative effects of the herbal medication are attributed to its stimulation of cell proliferation, neurite growth, synaptogenesis, and protection against hippocampal damage caused by global ischemic brain injury [14,16]. MLC901 possesses significant biological effects, including antioxidant and anti-inflammatory activities, modulation of immune responses, and neuroregenerative and neuroprotective properties. 

Studies have demonstrated that Growth-associated Protein 43 (GAP43), synthesized in growth cones, plays a crucial role in guiding and elongating growing axons [17]. GAP43 also referred to as neuromodulin, is involved in extracellular nerve cell growth, synapse development, formation, and the regeneration of nerve cells [18]. GAP43 is recognized as a marker indicating axon regeneration and the formation of new connections in nerve cells [12,19]. Changes in Glial Fibrillary Acidic Protein (GFAP) expression reflect the presence of reactive astrocytes. Elevated GFAP expression indicates the degree of astroglial reactivity following a spinal cord lesion [20].

The PI3K/AKT signaling pathway plays a crucial role in the pathology of spinal cord injury (SCI). Activating this pathway can help delay the inflammatory response, inhibit the formation of glial scars, and support the recovery of neurological functions [12,21]. Protein kinase B (AKT), a highly conserved serine/threonine kinase, is a key component of the PI3K signaling pathway. AKT exists in three isoforms: AKT1, AKT2, and AKT3. Through a phosphorylation cascade regulated by lipids and protein phosphatases, AKT influences various downstream effectors, including FOXO, mTOR, and GSK3β. This cascade plays a critical role in managing numerous cellular processes, such as cell growth, proliferation, survival, genomic stability, glucose metabolism, and neovascularization [21]. The activation of the PI3K/AKT signaling pathway can promote autophagy. Insulin-like Growth Factor 1 (IGF-1) inhibits autophagy by activating the PI3K/AKT signaling pathway following SCI. This activation has a protective effect on nerves in SCI rats and supports the recovery of neural function [21]. Numerous studies have reported that the PI3K/AKT signaling pathway plays a role in regulating neuronal cell proliferation and differentiation [22], myelination [23], and axon regeneration during neuronal differentiation [24].

This study aimed to induce spinal cord injury (SCI) in rats using the calibrated forceps compression method, followed by treatment with MLC901. The primary outcome measured was the functional improvement in MLC901-treated SCI rat models compared to untreated SCI rats. Administration of MLC901 promoted functional recovery in the rats. Histological and pathological analyses demonstrated that MLC901 minimized neuronal tissue damage following compression-induced SCI. Additionally, MLC901 treatment inhibited apoptosis and promoted neurogenesis by activating the PI3K/AKT/GSK3 signaling pathway.

## 2. Methodology

Details of the methodology are provided in Supplementarry Data S1. The spinal cord injury (SCI) rat model was created using calibrated forceps compression (mechanical method) in adult male Sprague Dawley rats. Following SCI induction, the injured rats were treated with 10 mg/kg/day of MLC901 for 28 days. Throughout the 28 day treatment period, the animals were assessed for locomotor, sensory, and electrophysiological functions. After 28 days of injury and treatment, both MLC901-treated (T) and untreated (UT) rats were sacrificed for histological and immunohistochemical studies. 

### 2.1. Calibrated Forceps Compression Injury

**Figure 1:**
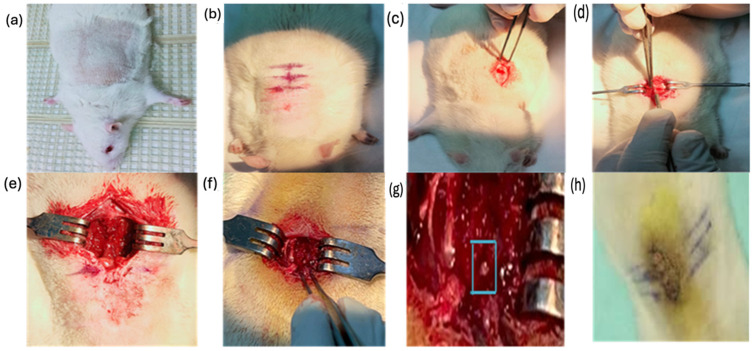
Diagrammatic representation of the step-by-step procedure for creating a mechanical spinal cord injury (SCI) model using the calibrated forceps compression method. (**a**) Sublime Animal Position: The animal is positioned in a prone orientation on the surgical table, ensuring stability and access to the spinal region. (**b**) Marking T10, T12, and T13 vertebra: Identification of the vertebrae to be targeted and marked for precise surgical intervention. (**c**) subcutaneous cut: An incision is made through the skin to gain access to the underlying tissues. (**d**) Removing Muscles: The overlying muscles are carefully removed to expose the spinal column while minimizing damage to surrounding tissues. (**e**) Exposing Spinal Cord: The spinal column is accessed, providing visibility to the spinal cord. (**f**) Removing T12 Vertebrae: The T12 vertebra is removed to allow direct compression of the spinal cord. (**g**) Compression of the Spinal Cord for 15 Sec: The spinal cord is compressed using calibrated forceps for a precise duration to induce injury, the blue box indicates spinal cord exposure and the compression site. (**h**) Wound Closing (Suture of Tissue and Skin): The surgical wound is closed with sutures, including tissue layers and skin, to complete the procedure, *n* = 6: Indicates the number of animals used for this procedure.

The compression injury was performed using the method described by McDonough et al. in 2015 [25]. First, a pair of fine-tipped Dumont #5 forceps was used to remove attached tissue from the lamina to expose the spinal cord. Small Vanna scissors were then inserted along the dorsolateral side of the vertebra, just beneath the lamina. The scissors were carefully used to cut through the lateral side of the vertebral lamina, and the bone was removed on the other side of the vertebra. No pressure was applied to the exposed spinal cord, and any bleeding was gently controlled with a Q-tip or surgical sponge. After making the incisions, the dorsal aspect of the vertebra was lifted, and any tissue attachments were cleared. Next, rongeurs or laminectomy forceps were placed parallel to the exposed spinal cord. The arms of the Dumont #5 forceps were slowly inserted into the epidural space on either side of the spinal cord, ensuring that the tips of the forceps reached the floor of the vertebral canal. The spinal cord was then compressed until the spacers at the tip of the forceps met, leaving a 1 mm gap between them [25]. Compression was maintained for 15 s, after which the force was gently released. A sterile saline solution was used to restore homeostasis before closing the wound. A diagrammatic representation of the mechanical SCI model using the calibrated forceps compression method is shown in Figure 1. Both injury groups were compared to the sham group to assess the severity of the injury, and the treated (T) and untreated (UT) groups were compared to evaluate the regenerative potential of MLC901.

### 2.2. MLC901 Treatment and Study Groups

Eighteen rats ranging between 300 and 400 g weight were randomly assigned to three groups (*n* = 6/group): (1) sham or healthy (H), in which animals underwent laminectomy surgery without any compression and treatment; (2) UT compression injury animals, in which rats underwent calibrated compression laminectomy with a lesion but without treatment for 28 days; the UT group mean vehicle control, the animal was administered with an equal amount of normal saline solution without the drug and (3) T rats, in which rats underwent calibrated compression laminectomy and received 10 mg/kg/day oral daily dose of MLC901 for 28 days. All calibrated compression-injured rats underwent compression for 15 s with a similar magnitude of force, which was confirmed by the pilot study conducted before this study.

### 2.3. Statistical Analysis

Statistical analysis was conducted using SPSS version 19.0 software (IBM Corp., Armonk, NY, USA). Results are presented as mean ± SEM, with values as specified in each figure legend. The symbol *n* represents the number of animals used and analyzed. To compare two independent groups, parametric values were assessed using Student’s *t*-test. For multiple comparisons, a one-way Analysis of Variance (ANOVA) followed by Dunnett’s post hoc test was employed. The significance level was set at *p* < 0.05.

## 3. Results

### 3.1. Effect of MLC901 Treatment on the General Health, Body Weight, and Autonomic Functions

**Figure 2:**
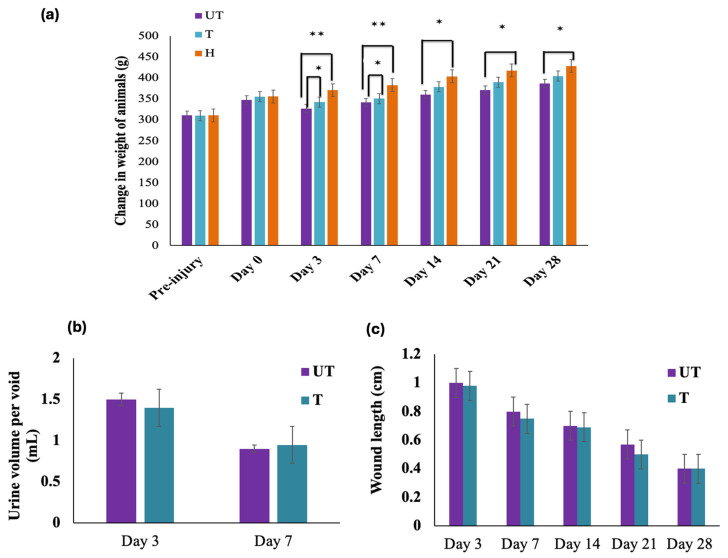
Health evaluation after compression injury. (**a**) Changes in body weight following spinal cord injury: Injured rats initially lost more body weight on Days 3 and 7 than sham (H) rats, but later began gaining weight. Treated (T) rats lost less weight compared to untreated (UT) animals on Days 3 and 7 (mean ± standard deviation [SD]; one-way analysis of variance [ANOVA] with post hoc test, * *p* < 0.05, ** *p* < 0.01) (*n* = 6). (**b**) Urine volume per void: Manual bladder voiding by the experimenter was required for 1 week (Day 7) post-SCI, after which spontaneous recovery began. No significant difference was observed between UT and T rats. Data were analyzed using Student’s *t*-test for non-parametric and unpaired comparisons (*n* = 6). (**c**) Length of the incision area: The length of the incision area was measured up to Day 28 post-injury. Wound recovery continued until Day 28, with no significant difference observed between UT and T rats. Data were analyzed using Student’s *t*-test for unpaired comparisons (*n* = 6).

The general health of the animals was not compromised following compression injury. No adverse effects, such as sickness behavior or tissue reactions to the MLC901 treatment, were observed. During the first week post-injury, animal body weight was closely monitored twice daily for the first 3 days, and then once daily for the remaining 4 days of the week. If an animal was found not eating or drinking properly, Ringer’s lactate solution was administered to maintain its body weight in both the treated (T) and untreated (UT) groups. While body weight initially decreased following compression injury, it gradually recovered over time (Figure 2a).

Body weight in all rats typically dropped by 10–15% during the first 7 days post-injury (*p* < 0.001) and remained lower (*p* < 0.05) on days 14, 21, and 28 compared with the sham group (H) (Figure 1a). The T rats showed faster recovery compared to the UT group, with an average weight gain of about 8% on days 3 and 7 (*p* < 0.05), which then increased to 2% per week, with no significant difference between the T and UT groups thereafter (Figure 2a). MLC901 treatment significantly affected the relative change in body weight in the T group compared to the UT group (*p* < 0.05) during the first-week post-injury. Post hoc analysis revealed that T rats initially lost significantly less weight than UT rats and continued to gain significantly more weight compared to the UT group (Figure 2a).

After SCI, the animals required assistance with bladder voiding. The volume of manually expelled urine was measured, and it was found that interventions did not significantly impact the return of spontaneous bladder control. Both the treated (T) and untreated (UT) groups regained spontaneous bladder control within 1 week post-SCI (Figure 2b). The spinal cord incision showed gradual recovery over time, and wound length measurements indicated continued healing until Day 28 in both UT and T rats, with no significant difference between the groups (Figure 2c).

Unexpectedly, two rats in the UT group developed leg ulcers, which appeared after 2 weeks and were treated with a wound care solution. The 4 week mark healed these ulcers.

### 3.2. MLC901 Facilitates Locomotor Functional Recovery and Ameliorates Tissue Damage Following SCI

**Figure 3:**
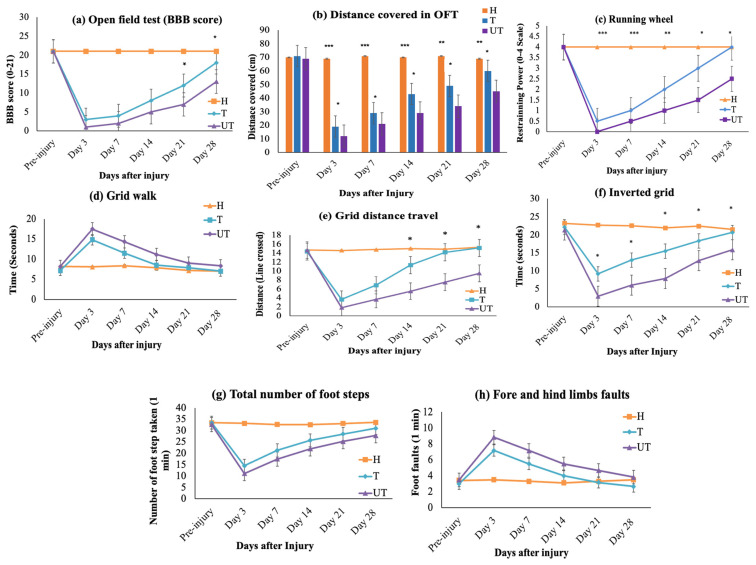
Assessment of locomotor function following MLC901 treatment (**a**) Basso, Beattie, and Bresnahan (BBB) scores, (**b**) distance traveled in the open field test (OFT), (**c**) performance in the running wheel test, (**d**) grid walk assessment, (**e**) grid distance traveled, (**f**) inverted grid (grip strength test), (**g**) total number of footsteps taken, and (**h**) fore- and hind-limb faults. Assessments were conducted on Day 0 (pre-injury) and Days 3, 7, 14, 21, and 28 post-injuries. Data are presented as means with error bars indicating the standard error of the mean (*n* = 6). Statistical analysis was performed using one-way ANOVA followed by post hoc tests. Treated (T) rats demonstrated significant recovery and improvement compared to controls. Statistical significance is indicated as * *p* < 0.05, ** *p* < 0.01, and *** *p* < 0.001 for comparisons among the three groups (sham [H], treated [T], and untreated [UT]).

With meticulous precision, the rat’s locomotor recovery and motor ability were analyzed using the Basso, Beattie, and Bresnahan (BBB) score, which focuses on parameters such as step width, step length, and coordination between the forelimb and hindlimb. The BBB scores, assessed by two blinded observers, revealed a significant difference between the untreated (UT) and treated (T) groups (Figure 3a). All injured rats exhibited signs of paraplegia and showed significantly lower BBB scores compared to the sham (H) group (Figure 3a).

The UT SCI group displayed uncoordinated footprints, hind-leg dragging, narrow step width, reduced distance between forelegs, and an elongated gap between the forelegs and hindlegs (Figure S1). On the third day post-injury, the BBB score for UT rats dropped to 2 points and gradually increased until Day 28, whereas T rats demonstrated faster recovery. On Days 21 and 28 post-injury, T rats showed statistically significant improvement in locomotor functions, achieving mean BBB scores of 14 and 19 points, respectively. In contrast, the UT rats’ mean BBB scores remained at 9 and 13 points. Thus, the T rats exhibited a statistically significant improvement compared to the UT rats on Days 21 and 28 (*p* < 0.05) (Figure 3a and Figure S1).

The distance traveled by rats in the open field test (OFT) was measured over a 5 ± 1-min period. Significant differences were observed between the sham (H) and injury groups (T and UT), indicating successful SCI induction (*p* < 0.001 and *p* < 0.01) (Figure 3b). On Days 3, 7, 14, 21, and 28, T rats traveled a greater distance in the open field compared to the UT group (*p* < 0.05) (Figure 3b). 

To further assess hind-limb motor function and coordination, additional tests were conducted, including the running wheel, grid walk, and grip strength tests. Significant differences were observed between the MLC901-treated (T) and UT groups. In the running wheel test, both UT and T rats showed a complete loss of restraining power on Day 1 post-injury, with a significant reduction in comparison to the H group (*p* < 0.001) (Figure 3c). From Day 3 to Day 14, T rats did not show significant differences compared to UT rats, but on Days 21 and 28, T rats exhibited a significant improvement in restraining power (*p* < 0.05) compared to UT rats (Figure 3c).

In the grid walk test, both injured groups demonstrated significant impairments in walking strength, with a higher number of foot faults (hind limbs) observed (Figure 3d,e). Although there was no significant difference in the time taken to cross the grid between UT and T groups (Figure 3d), T rats showed significant improvements in the number of lines crossed and foot faults on Days 14, 21, and 28 compared to UT rats (*p* < 0.05) (Figure 3e). Grid walk analysis also revealed that T rats performed significantly better than UT rats in terms of the total number of footsteps taken and foot faults within a 1 min assessment (*p* < 0.05) (Figure 3g,h).

Grip strength testing using the inverted grid method showed that both injured groups lost significant strength to hold the grid in the inverted position due to hind limb paralysis (Figure 3f). However, with progressive treatment, T rats demonstrated greater holding time compared to UT rats, with significant improvements observed on Days 7, 14, 21, and 28 post-SCI, indicating enhanced grip strength with MLC901 treatment (Figure 3f).

In addition to motor assessments, sensory function was evaluated using the hot spatula and cold ethanol tests. T rats showed faster recovery compared to UT rats in withdrawal behavior scores (Figure S2). 

### 3.3. Somatosensory Evoked Potentials (SEPs) Improved after MLC901 Treatment in Mechanically Injured Rats

**Figure 4:**
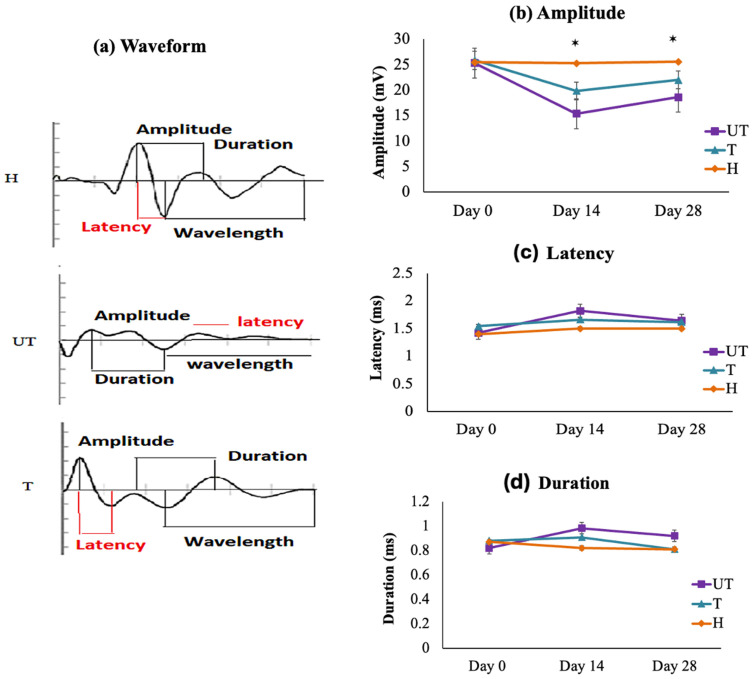
Somatosensory Evoked Potential (SEP) Analysis. (**a**) Waveform: SEPs were obtained by stimulating the left hindlimb in sham (H), untreated (UT), and treated (T) rats with moderate compression injury on Day 0 (pre-injury), Day 14, and Day 28 post-injury. The waveform data show the characteristic peaks and changes over time. (**b**) Amplitude: Amplitude of SEPs was significantly reduced in UT rats compared to sham controls, with treated (T) rats showing a significant improvement. Statistical significance (*p* < 0.05) was observed between UT and T rats on Day 14 and Day 28. (**c**) Latency: The latency period of SEPs was significantly increased in injured rats compared to sham controls, with no significant differences between UT and T rats at Days 14 and 28. (**d**) Duration: The duration of SEPs, inversely related to nerve conduction velocity, was longer in UT rats compared to T rats. No significant differences were found between groups at Days 14 and 28. Data are expressed as mean ± standard deviation (SD) and were analyzed using one-way analysis of variance (ANOVA) followed by post hoc tests (*n* = 6). Statistical significance is indicated by * *p* < 0.05.

The somatosensory evoked potentials (SEPs) are characterized by four prominent peaks: an initial peak at 5 milliseconds (ms), followed by two downward positive and two upward negative peaks in an alternating pattern. Post-injury, the amplitudes of these peaks are reduced, and there is a corresponding decrease in the average SEPs nerve conduction velocity. Electrophysiological results correlated with locomotor outcomes. The sham (H) group exhibited stable SEPs waveforms over time (Figure 4a). In contrast, the SEPs waveform in both treated (T) and untreated (UT) rats were absent immediately after SCI, with no waveform detected even 30 min post-injury. By Day 14, UT rats showed severe motor dysfunction in their hind limbs, as indicated by decreased amplitude and a slight increase in duration and latency compared to the T group (Figure 4a–d).

Amplitude, duration, and latency were recorded for UT and T rats at pre-injury (Day 0), and on Days 14 and 28 post-injuries. For a representative rat with no laminectomy (no injury), the amplitude of averaged SEPs signals on Days 0, 14, and 28 is shown in Figure 4b. The H group maintained a stable waveform over time, confirming that anesthesia did not adversely affect SEPs signals. In contrast, both UT and T rats showed a statistically significant (*p* < 0.05) reduction in amplitude on Day 14 compared to the H group. The mean amplitude for UT rats was 18.86 ± 1.52 mV on Day 14 and 21.5 ± 0.42 mV on Day 28, while for T rats, it was 20.63 ± 0.31 mV on Day 14 and 26.02 ± 1.14 mV on Day 28 (Figure 4b), respectively. 

Latency significantly increased (*p* < 0.05) in injured rats compared to the H group. On Day 14, the mean latency was 1.82 ± 0.73 ms for UT rats and 1.76 ± 1.46 ms for T rats. By Day 28, the latency was 1.74 ± 0.32 ms for UT rats and 1.64 ± 0.14 ms for T rats (Figure 4c). Duration, which inversely relates to nerve conduction velocity, was longer in UT rats compared to T rats. On Days 14 and 28, the mean duration for UT rats was 1.02 ± 0.47 ms and 0.899 ± 0.35 ms, respectively, while for T rats, it was 0.812 ± 0.17 ms on Day 14 and 0.875 ± 0.63 ms on Day 28 (Figure 4d).

### 3.4. The Lesion Size, Tissue Loss, and Cystic Cavitation after Mechanical Injury and Neuroprotective Activity of MLC901

**Figure 5:**
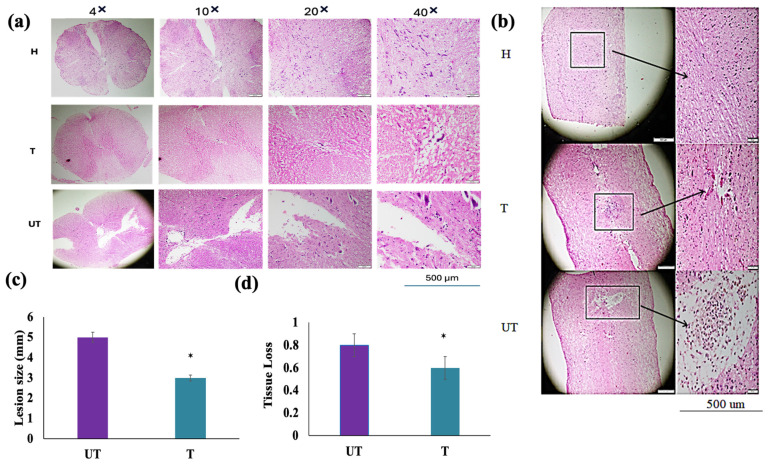
Histological analysis of spinal cord: (**a**) Transverse Section: Transverse spinal cord sections stained with hematoxylin and eosin (H&E) at 4 weeks post-injury. This view highlights the extent of tissue damage and demyelination in untreated (UT), treated (T), and sham (H) rats. Images are captured at 4× magnification with a scale bar of 500 µm. Significant differences in tissue integrity and lesion characteristics are evident between groups. Sham (H) rats show normal spinal cord morphology, while UT rats display extensive tissue damage and large cystic cavities. Treated (T) rats show reduced lesion size and less severe tissue degeneration compared to UT rats. (**b**) Longitudinal Section: Longitudinal spinal cord sections stained with H&E, provide a detailed view of demyelination and histopathological features across the length of the injury. Images are taken at 10× magnification with a scale bar of 500 µm. Similar to the transverse sections, UT rats exhibit pronounced tissue loss and hemorrhagic foci, while T rats demonstrate reduced tissue damage and smaller lesions. Sham (H) rats present with normal spinal cord structure. (**c**) Relative Tissue Loss: Quantitative analysis of tissue loss in the center of the lesion, normalized to spinal cord sections from sham (H) rats without lesions. Bars represent the means and standard deviation (SD) of tissue loss measurements. Significant differences are observed between treatment groups, with T rats showing less tissue loss compared to UT rats (*p* < 0.05). Data were analyzed by one-way analysis of variance (ANOVA) with the Dunnett post hoc test. (**d**) Lesion Size: Measurement of lesion size in the center of the injury site, comparing untreated (UT), treated (T), and sham (H) rats. Bars represent the means and standard deviation (SD) of lesion size measurements. Statistical Significance: Significant reduction in lesion size in T rats compared to UT rats (*p* < 0.05 *). Data were analyzed using one-way ANOVA with the Dunnett post hoc test. These findings confirm that MLC901 treatment effectively mitigates spinal cord damage and supports tissue repair following mechanical compression injury.

After 28 days of treatment and locomotor assessment, the animal was sacrificed, and the intact spinal cord was isolated from all groups for hematoxylin and eosin (H&E) and Immunohistochemistry (IHC) staining (Figure S3). Figure 5 shows a histological analysis of Spinal Cord Damage Post-Injury. Figure 5a reflects spinal cord sections from sham (H), untreated (UT), and treated (T) rats that were stained with H&E after 28 days of treatment. The UT group exhibited large cystic cavities and severe damage at the lesion site, characterized by substantial loss of the ventral gray matter horns and the lateral white matter funiculus. In contrast, T rats treated with MLC901 showed significantly reduced cystic cavitation and less severe tissue damage, indicative of neuroprotective repair. Figure 5b describes histological damage observations: UT rats demonstrated extensive hemorrhagic foci and progressive necrosis within the gray matter, reflecting pronounced nerve degeneration and tissue loss. Conversely, T rats had smaller microcystic structures and reduced hemorrhage, signifying improved tissue preservation and a smaller lesion size. Figure 5c showed quantitative measurements of lesion size and tissue loss showing that MLC901 treatment significantly reduced both total lesion size and tissue loss compared to the UT group. This indicates effective repair and neuroprotection provided by MLC901. Figure 5d represents the inflammatory response: both UT and T rats showed signs of inflammatory response, including the presence of erythrocytes and neutrophils at the lesion site. However, these inflammatory markers were more prominent in UT rats, suggesting a more severe inflammatory response compared to the treated group. The data underscore the effectiveness of MLC901 in mitigating spinal cord damage, reducing cystic cavitation, and preserving spinal cord tissue integrity.

### 3.5. Effect of MLC901 Treatment on GAP-43 and GFAP Expression

Following the 28 day treatment period, spinal cord sections were examined to assess the impact of MLC901 on astrocytic scar formation and the expression of Glial Fibrillary Acidic Protein (GFAP) and Growth-Associated Protein 43 (GAP-43). The following analyses were performed. 

#### 3.5.1. Growth-Associated Protein 43 (GAP-43) Expression

**Figure 6:**
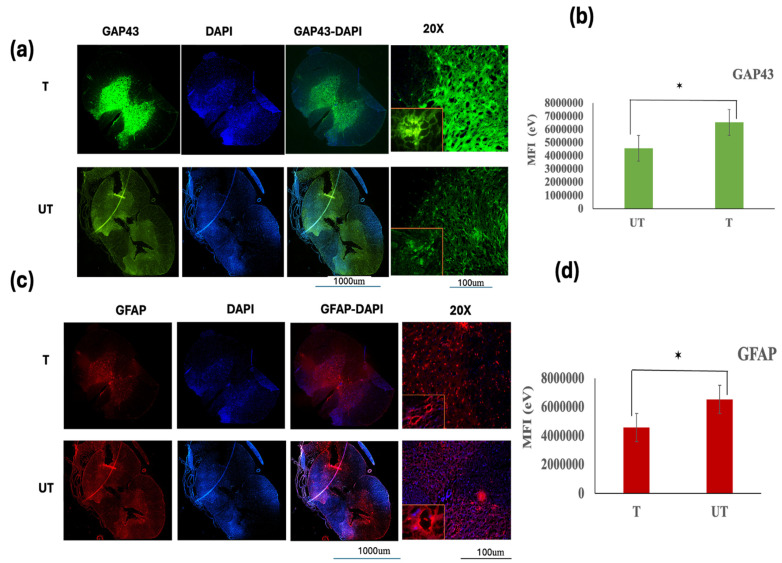
Comparison of GAP-43 and GFAP expression 28 days post-compression spinal cord injury with MLC901 treatment. (**a**,**b**) GAP-43 Expression: Immunofluorescence staining revealed that the intensity of Growth-associated protein 43 (GAP-43, green) was significantly higher in MLC901-treated (T) rats compared to untreated (UT) rats 28 days post-injury ((**a**,**b**); *p* < 0.05 *). This increased GAP-43 immunoreactivity indicates enhanced neurogenesis and axonal growth in the T group. (**c**,**d**) GFAP Expression: Conversely, the intensity of Glial fibrillary acidic protein (GFAP, red) immunoreactivity was notably lower in the T rats compared to the UT rats ((**c**,**d**); *p* < 0.05 *). Reduced GFAP staining suggests that MLC901 treatment effectively mitigates astrocytic scar formation and inflammation, contributing to a more favorable environment for neuroprotection and recovery. Nuclei Staining: Nuclei are stained with DAPI (blue), which helps to visualize cell bodies in the spinal cord sections. The higher magnification images (20×) on the right side of the figures showed morphological changes and detailed regions of demyelination with double staining for GAP-43-DAPI and GFAP-DAPI, highlighting the distinct areas of neurogenesis and astrocytic response. Scale bars are 1000 µm and 100 µm, providing context for the images’ magnification. Data are expressed as mean ± standard deviation (SD). Statistical significance was determined using Student’s *t*-test (*n* = 6). Significance is indicated as * *p* < 0.05. These findings support that MLC901 treatment enhances neurogenesis while reducing astrocytic scar formation, contributing to improved functional recovery following spinal cord injury.

GAP-43 is a protein associated with neuronal growth and regeneration. Its expression levels were assessed to evaluate neuronal repair and axonal regeneration. Immunofluorescence staining revealed a significantly stronger fluorescence intensity for GAP-43 in the treated (T) rats compared to the untreated (UT) rats at 4 weeks post-injury (Figure 6a,b). This indicates that MLC901 treatment enhanced neuronal growth and axonal regeneration. The mean fluorescence intensity (MFI) of GAP-43 in T rats was statistically significant (*p* < 0.05) compared to UT rats, demonstrating the efficacy of MLC901 in promoting neuronal repair. A strong positive correlation was observed between BBB scores and GAP-43 intensity in T rats, suggesting that improved locomotor recovery is associated with increased GAP-43 expression (Figure 6b).

#### 3.5.2. Astrocytic Scar Formation and GFAP Expression

Astrocytic scars, characterized by increased GFAP expression, were evaluated using immunohistochemistry. GFAP is a marker for astrocytes and is often upregulated in response to spinal cord injury, contributing to scar formation and tissue repair. GFAP immunofluorescence staining showed the presence of GFAP-positive astrocytic processes, indicating inflammatory responses and glial scar formation following compression injury in both T and UT rats (Figure 6c,d). The MFI data for GFAP demonstrated that MLC901 treatment significantly reduced GFAP expression compared to the UT group, suggesting that MLC901 effectively inhibits glial scar formation. The MFI of GFAP in T rats was significantly lower than in UT rats (Figure 6d).

#### 3.5.3. Double Staining Analysis

To further assess the effects of MLC901 on neuronal and glial markers, double staining was performed on transverse spinal cord slices using GAP-43, GFAP, and DAPI. The results presented in Figure S4 illustrate the differences in expression between T, UT, and sham (H) rats, highlighting the neuroprotective effects of MLC901 and its role in reducing glial scar formation and promoting neuronal growth.

Data are presented as mean ± standard deviation (SD). Statistical significance was determined using one-way analysis of variance (ANOVA) followed by post hoc tests (*n* = 6). Statistical significance is denoted as * *p* < 0.05.

### 3.6. MLC901 Promoted Neurogenesis and Activated the PI3K/AKT/GSK-3β Signaling Pathway

#### 3.6.1. PI3K/AKT Signaling Pathway Activation

**Figure 7:**
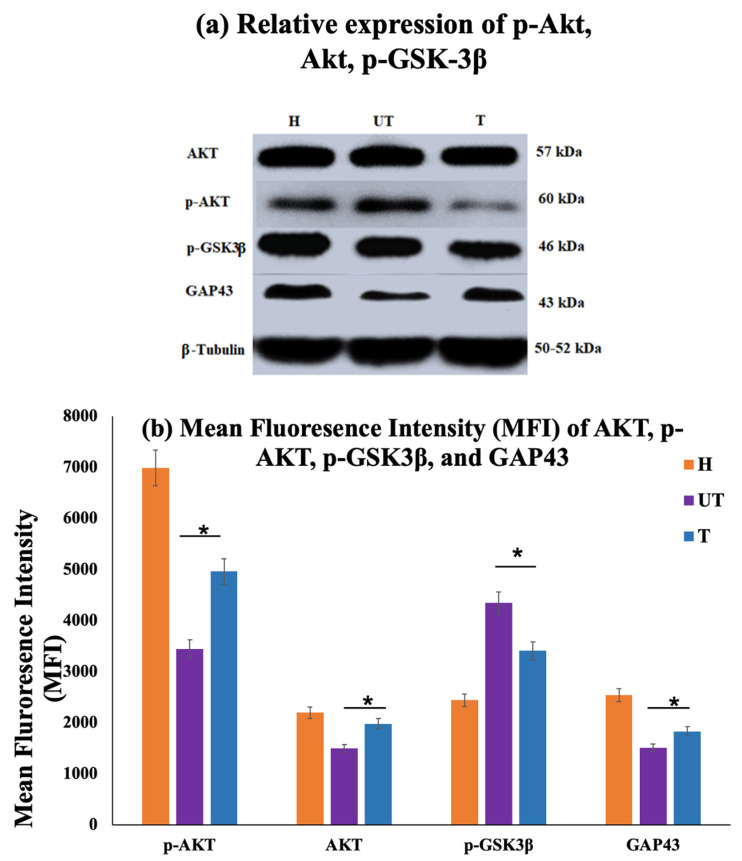
MLC901 activated the PI3K/AKT/GSK-3β signaling pathway. (**a**) Western Blot Analysis: To assess the relative expression levels of p-AKT, AKT, p-GSK-3β, and GAP-43 proteins in spinal cord tissues from sham (H), treated (T), and untreated (UT) rats with β-Tubulin as a loading control. (**b**) Mean Fluorescence Intensity (MFI): To measure the expression levels of p-AKT/AKT, p-GSK-3β, and GAP-43 (neurogenesis marker) in spinal cord tissues of sham (H), treated (T), and untreated (UT) rats. (i) p-AKT/AKT Ratio: T rats showed higher expression compared to UT rats, indicating enhanced activation of the PI3K/AKT pathway. (ii) p-GSK-3β expression: UT rats showed higher expression compared to T rats, suggesting that MLC901 treatment inhibits GSK-3β activity and reduces apoptosis. (iii) GAP-43 Expression: Significantly higher expression in T rats compared to UT rats (*p* < 0.05), indicating that MLC901 promotes neurogenesis. Statistical Analysis: Data are presented as mean ± standard deviation (SD) (*n* = 6/group). Statistical significance was determined using a one-way analysis of variance (ANOVA), followed by a post hoc test. * *p* < 0.05 indicates significant differences between T rats and the UT SCI group.

The PI3K/AKT pathway is closely related to neurogenesis after SCI. To investigate the underlying mechanism by which MLC901 promoted neuroprotection, the *p*-AKT and AKT levels were assessed; the levels of phosphorylated AKT (p-AKT) and total AKT (AKT) were assessed in spinal cord tissues from treated (T) and untreated (UT) rats. The ratio of p-AKT to AKT was significantly higher in the MLC901-treated (T) group compared to the UT group, indicating enhanced activation of the PI3K/AKT pathway (Figure 7a,b).

#### 3.6.2. Neurogenesis and GAP-43 Expression

The expression of Growth-associated protein 43 (GAP-43), a marker of neurogenesis and axonal growth, was evaluated. The T group showed significantly higher GAP-43 expression than the UT group (Figure 7b). This increase in GAP-43 suggests that MLC901 enhances neurogenesis following SCI (Figure 7a,b).

#### 3.6.3. GSK-3β and Apoptosis

The levels of phosphorylated GSK-3β (p-GSK-3β) were measured to assess the anti-apoptotic effect of MLC901. The expression of p-GSK-3β was significantly lower in the UT group compared to the T group. This decrease indicates that MLC901 treatment reduces apoptosis through inhibition of GSK-3β (Figure 7a,b).

#### 3.6.4. Neuroprotection and Neurogenesis

MLC901 treatment leads to increased activation of the PI3K/AKT pathway and higher expression of GAP-43, suggesting that it promotes neuroprotection and neurogenesis in SCI rats.

#### 3.6.5. Anti-Apoptotic Effects

The reduction in p-GSK-3β expression following MLC901 treatment highlights its role in inhibiting apoptosis and protecting neuronal cells through the PI3K/AKT/GSK-3β signaling pathway. These findings support the hypothesis that MLC901 enhances neuroprotection and promotes recovery after SCI by activating key signaling pathways involved in neurogenesis and cell survival.

### 3.7. Assessment of Hepatotoxicity and Nephrotoxicity Associated with MLC901 Treatment

**Figure 8:**
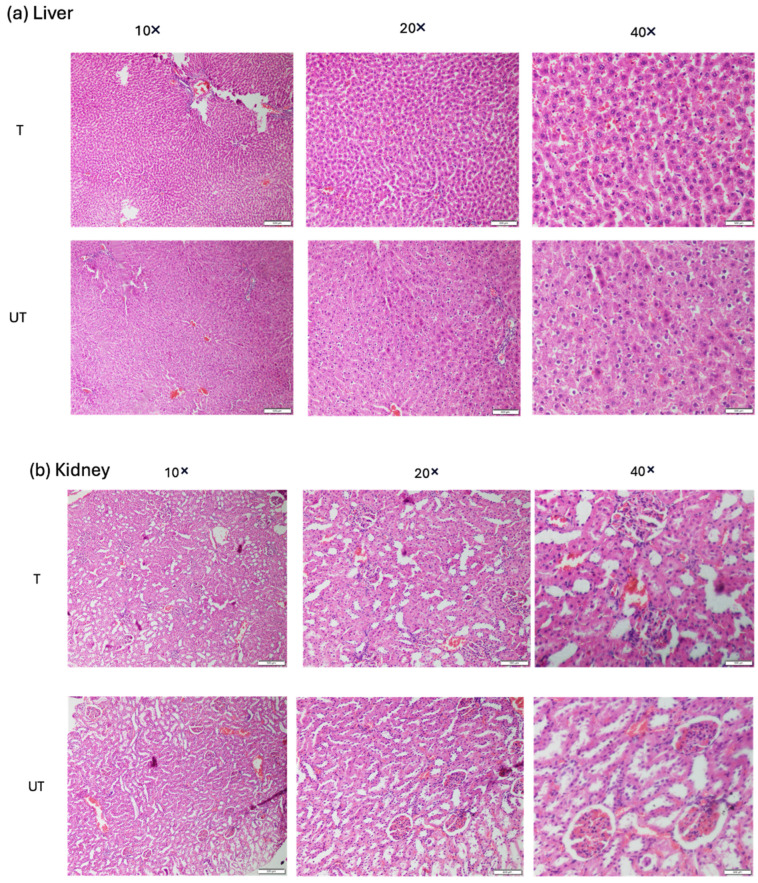
Histopathological Evaluation of Liver and Kidney Tissues. (**a**) Hepatic Tissue (Liver): Hematoxylin and eosin (H&E) staining of liver tissue sections to assess potential hepatotoxicity associated with MLC901 treatment, observed under different magnifications (10×, 20×, and 40×), using scale bars 50, 100, and 500 µm. The images showed no sign of sinusoidal dilatation (enlargement of the hepatic capillaries), necrosis, hemorrhage, or congestion observed in treated (T) rats associated with MLC901 treatment, in liver tissue. MLC901 treatment did not induce hepatotoxicity after 28 days, indicating the liver remained healthy under the treatment conditions, *n* = 6. (**b**) Renal Tissue (Kidney): Hematoxylin and eosin (H&E) staining of kidney tissue sections to evaluate potential nephrotoxicity associated with MLC901 treatment, observed under different magnifications (10×, 20×, and 40×), using scale bars 50, 100, and 500 µm. The images showed no vacuolization in tubular cells, focal necrosis, or hemorrhage observed in the kidney tissue of T rats. MLC901 treatment did not induce nephrotoxicity after 28 days, confirming that the kidney function remained intact, *n* = 6. Both liver and kidney tissues in T rats showed no adverse effects such as hepatotoxicity or nephrotoxicity, suggesting that MLC901 is safe for these organs after 28 days of treatment, UT represents untreated rats without MLC901 treatment and receiving normal saline.

The liver and kidney are directly involved in drug metabolism and the excretion of the drug from the body. The liver and kidneys play a central role in drug metabolism, which makes them susceptible to toxic injury. Several herbal drug compositions have beneficial effects, and can potentially improve health conditions in severe diseases, but they may have the tendency to exert toxicity to the body. In this study, histopathological and biochemical changes due to MLC901 treatment in the liver and kidney were assessed in both T and UT rats. The results obtained in Figure 8a showed no sinusoidal dilatation (enlargement of the hepatic capillaries), necrosis, hemorrhage, or congestion observed in T rats with MLC901 treatment. Additionally, no perivenular necrosis or hemorrhage was observed in T rats, ensuring that MLC901 did not induce hepatotoxicity following 28 days of treatment. Similarly, in the histopathologic finding of kidney slices, no vacuolization in tubular cells (the formation of vacuoles or vacuole-like structures, within or adjacent to cells), focal necrosis, and hemorrhage in the T rats were observed (Figure 8b), ensuring no nephrotoxicity associated with MLC901 treatment. Only mild inflammation was observed (Figure 8a) in liver slices.

## 4. Discussion

The calibrated forceps mechanical injury in the male Sprague Dawley rats displayed major signs of motor dysfunction and sensory impairment. The behavioral changes in terms of limited eating and drinking were observed, until 7 days post-injury, along with urinary retention. These problems were easily monitored and treated, without any detrimental effect during the study. The urinary retention also recovered within 7 days post-injury. In this study, the UT rats at day 3 showed the lowest BBB scores of 2 and 3 only, which indicated a moderate spinal cord injury stage of SCI. It was also observed that the T rat showed improvement in locomotion activity and reached a mean score of 19 after 28 days, while the UT rat reached a mean score of only 13 after 28 days. Two blinded observers rated the behavior from individual joint movements of the hind limb to plantar stepping, to coordinated walking, and finally the subtler behaviors of locomotion, such as paw position, trunk stability, and tail position (Figure 3 and Figure S1). While the control rats showed a mean score of 21 throughout the experiment, the score of 21 indicated “Consistent plantar stepping and coordinated gait” as presented previously [26,27].

The T rats showed a short lag phase between the first 3 days of treatment, followed by a more rapid phase of recovery between day 4 to day 14, after that a functional plateau between day 15 to day 28 was observed. The pattern of BBB score achieved up to day 28 was found quite like previously reported studies with the same severity of injury [28]. Other locomotor assessments, such as the running wheel are considered an important parameter to assess the strength and coordination in rats after SCI [29,30]. It is primarily utilized to assess motor deficits in animal models of Multiple Sclerosis, Parkinson’s Disease, hyperactivity in genetic models of attention-deficit-hyperactivity disorder, and spinal cord injury [31]. The quantification of wheel running behavior has typically focused on the restraining time measurement between different groups [31].

Similarly, the grid walk test is an important laboratory behavior assessment that helps evaluate the gait analysis of different study groups. The grid walking test was utilized previously, to investigate sensorimotor deficits and motor incoordination in rats following spinal cord injury (SCI) [32]. Researchers employed a method where rats were expected to place their limbs accurately in specific locations. Each time a rat’s paw missed its intended placement, it was recorded as a foot fault. The total number of errors and foot faults was counted during the grid walking from one end of the grid to another end. The greater number of foot faults reflected defects in motor coordination. It was also studied previously and reported that SCI rats showed higher foot faults than healthy rats [32]. The inverted grid test is another excellent tool that helps to evaluate limb power and motor coordination [33]. The T rats showed a significant increase in inverted grid holding time than the UT rats. These findings proved that treatment with MLC901 improved motor coordination reduced the number of foot faults, improved the restraining power, and overall promoted locomotor coordination in injured rats.

The low amplitude and increase in latency and duration emphases on the slow nerve conduction velocity showed a demyelinating lesion, whereas the low amplitude value reflected axonal degeneration [34]. In this study, we found that no nerve conduction was observed just after injury in both injured rat groups, then slow nerve conduction was observed on day 14 and day 28 in injured rats. It was also found that the T group showed better nerve conduction velocity than UT rats on day 14 and day 28.

The hemorrhage and cavitation in UT rats were higher than in T rats, while cavity formation was reduced with the MLC901 treatment. The greater presence of inflammatory cells such as the presentation of erythrocytes and neutrophils in both injured rat groups was observed than in healthy rats, showing nerve degeneration caused by calibrated forceps compression injury in rats. The decrease in lesion size and tissue loss following MLC901 emphasizes the neuroprotective effect of treatment toward pathological cascade events. MLC901 as an oral drug for 28 days showed no adverse effects on hepatic and nephrotic tissue, and the H& E images showed no sign of degeneration and hemorrhage associated with the treatment of MLC90. Hence, MLC901 was found to be a safe treatment for 28 days. 

It has been demonstrated that the PI3K/AKT pathway plays a crucial role in controlling normal nerve cell function and balance and contributes to the recovery of subsequent neurologic deficits [21,35,36]. The expression of p-AKT and p-GSK3β showed an inverse relation. The higher expression of p-AKT reflected neurogenesis and the lower p-GSK 3β expression level indicated inhibition in cell apoptosis. Hence, the upstream expression of p-AKT (Thr308) in the T group with MLC901 reflected cell cycle regulation, neurogenesis, and prevention from GSK-3β-mediated phosphorylation and degradation. Similarly, the growth-associated protein 43 (GAP43) expression is another indicator of neurons and axon regeneration and demonstrates the new connection formation in nerve cells [17,26]. The upregulation of GAP43 expression in the T group indicated enhanced nerve cell growth in the damaged spinal cord. GAP-43 has been stated as a crucial protein associated with neurite growth. This study demonstrated that the expression level of GAP43 significantly increased with MLC901 treatment, suggesting enhanced neurogenesis. Even the immunohistochemistry results showed an increase in GAP43 expression (neuronal marker) and a decrease in GFAP (reactive astrocyte) in the T rats compared to the UT rats, further supporting the neuroregenerative and neuroprotective ability of MLC901.

MLC901 contains several active ingredients that are present in the herbal composition such as tetramethylpyrazine (*Rhizoma chuanxiong*), ferulic acid (*Radix angelicae sinensis*, *Rhizoma chuanxiong*), ligustilide, and butylidenephtalide (*Radix angelicae sinensis*, *Rhizoma chuanxiong*), astralagoside IV (*Radix astragali*), salvianolic acid B and tanshinone IIB (*Radix salvia miltiorrhizae*) that are known to possess neuroprotective, neuroregenerative and have anti-inflammatory properties. The MLC901 mechanisms of action can be explained as the reduction of tau phosphorylation at epitopes associated with neurofibrillary tangle formation [37], activating ATP-dependent potassium channels (KATP), and modulating neuroinflammation [38,39].

Although this study showed a promising impact of MLC901 treatment as a neuroregenerative drug, it is a combination of nine drugs, and not much information is known about the active constituent in each of the herbal drugs. The study on the effects of the defined chemical entities showed limitations of this study, but it should be included in future directions. To delve deeper into the potential molecular mechanisms responsible for the improved motor function observed in SCI rats treated with MLC901, further investigation could involve the addition of a PI3K or p-AKT inhibitor such as LY294002 for comparative purposes. This study would aim to assess the levels of PI3K/Akt pathway-related proteins following MLC901 treatment, including the ratios of p-AKT, AKT, and GSK-3β expression between treated (T) and untreated (UT) groups. Another factor to be considered for future research is to include other parameters such as oxidative stress, NOX2 level, and inflammatory mediators such as IL-1β, TNF-α, and IL-18. The neuro-inflammatory responses are also closely related to nerve injury and greatly impact neurogenesis and apoptosis. In future studies, the effect of MLC901 treatment on the neuro-inflammation and neuroinflammatory mediators can also be considered as a potential direction. 

Previous research indicates that spinal cord injury (SCI) is more prevalent among males, with 68.3% of cases involving males [40]. Over the past decade, age- and sex-standardized incidence rates for traumatic SCI have remained stable at 26.5 cases per 1,000,000 inhabitants (95% CI, 25.0–27.9), with an average age of 59.2 years. Additionally, female animals undergo estrous cycles that cause hormonal fluctuations, potentially introducing variability into experimental outcomes [41,42]. Given the higher prevalence of spinal cord injury (SCI) in males and to minimize outcome variability related to hormonal fluctuations, our study concentrated primarily on male subjects. While this approach helps control for certain variables, it introduces a minor bias and does not fully reflect clinical scenarios. Future research should address this limitation by including both male and female animals, ideally with a balanced ratio of approximately 68% male and 32% female subjects [40]. This would better replicate human SCI incidence and enhance the evaluation of new drug therapies.

## 5. Conclusions

This study explored the effects of MLC901 on spinal cord injury (SCI) using a forceps mechanical injury model. The study utilized a calibrated forceps mechanical injury model to simulate SCI in vivo. The model effectively demonstrated neurodegenerative outcomes consistent with SCI. This study also reflected that MLC901 treatment was shown to promote neurogenesis and inhibit neuronal apoptosis in rats with SCI. Significant improvements were observed in locomotor activity, sensory functions, and electrophysiological conduction in MLC901-treated rats. Histological analysis revealed reduced tissue damage and improved spinal cord integrity in treated rats. Activation of the PI3K/AKT/GSK-3β signaling pathway was identified as a crucial mechanism underlying the neuroprotective effects of MLC901. The data suggest that MLC901 effectively mitigates neurodegenerative effects by activating neuroprotective pathways. The study supports the potential of MLC901 as a potent neuroregenerative drug for SCI and potentially other neurodegenerative conditions. MLC901 represents a promising therapeutic strategy for managing traumatic spinal cord injury, offering potential benefits for improved recovery and function. In conclusion, MLC901 demonstrates significant potential as a therapeutic candidate for SCI, enhancing functional recovery and offering a viable approach for neuroprotection through the activation of key signaling pathways.

## Data Availability

The data presented in this study are available in the main text. Further information is available on request from the corresponding authors.

## Supplementary Materials

The following supporting information can be downloaded at: https://www.mdpi.com/article/10.3390/biomedicines12081920/s1, Data S1: detailed material and method section. The references [43,44,45,46,47] have been added in Data S1. Figure S1: BBB sub score, for i.e., jaw movement (0–7), paw placement (8–13), and toe clearance (14–21) between T and UT rats. Figure S2: Sensory function assessment for (a) hot, and (b) cold sensations. Figure S3: The photograph of the extracted spinal cord from different, UT and T rats. Figure S4: Immunohistochemistry (IHC) analysis of spinal cord. Video S1: The calibrated forceps compression injury model. Video S2: Treated (T) rats after 2 weeks of injury. Video S3: Untreated (UT) rats after 2 weeks of injury.
