# Supplementary material for: NeuroAiDTM-II (MLC901) Promoted Neurogenesis by Activating the PI3K/AKT/GSK-3β Signaling Pathway in Rat Spinal Cord Injury Models
Source: Biomedicines. 2024 Aug 21;12(8):1920. doi: 10.3390/biomedicines12081920 (PMC11352105; doi:10.3390/biomedicines12081920)
Supplement: Supplementary file 1 [file biomedicines-12-01920-s001.zip › biomedicines-3075000-supplementary.pdf]

Supplementary Data 1: Material & Methods

1. **Materials**

Animals

Male Sprague-Dawley rats (aged 6–8 weeks, weighing 300–400 g) were obtained from the Specific Pathogen Free (SPF) facility from the Animal Experimental Unit (AEU), Faculty of Medicine, University Malaya, Malaysia. They were housed in a standard light-controlled room (12:12-h light/dark cycle) and maintained at a constant temperature (25 ± 1 °C) and humidity (50–60%) for at least 1 week before the experiments. Food and water were freely available. All the experiments were conducted after getting approval from the University Kebangsaan Malaysia, Animal Ethical Committee (UKMAEC) with approval TEC/FP/2021/YOGESWARAN/27-MAY/1176-JUNE-2021-JUNE-2023.

Animal's Bedding and Feeding contain 15 bags of animal feed (Altromin NIH 31 M Diet), 2.5kg/vacuum packed bags, and 5 bags of 5-7mm thick corn cob laboratory animal bedding, 18kg/bag, that were bought from BIOSYSTEMS, BSC Malaysia Sdn Bhd.

Drugs, reagents, and antibodies

MLC901 was provided by Moleac Pte LTD, Singapore. KA or 2-Carboxy-3-carboxymethyl-4-isopropenylpyrrolidine was bought from Sigma-Aldrich, Malaysia, H and E Staining Kit (Hematoxylin and Eosin) purchased from Abcam USA, Glial fibrillary acidic protein (GFAP) anti-GFAP antibody and DAPI (4′,6-diamidino-2-phenylindole) were bought from ThermoFisher Scientific, USA and GAP43 (growth-associated protein) antibody was got from Cell Signalling UK. Isopropyl alcohol (70%), ketamine (90 mL), and xylazine (10 mL), were bought from the ilium (Malaysia), povidone (10% iodine) from Disinfecto Malaysia, antibiotic injection (Baytril (Enrofloxacin.100 mg/mL) from Bayer (Malaysia), tramadol injection (50 mg/mL) from DSN Pharma (Malaysia), ringer lactate solution was got from A. N. B Laboratories CO ltd (Thailand), 0.9% normal saline for injection (B Braun Medical industries Malaysia), wound clear solution (DyaMed Biotech), eye ointment (Kloraxin DSN Pharma SDN), pentobarbital (200mg/mL) (Vetoquinol Malaysia), Eosin-Y from and Hematoxylin-2 stain was bought from ThermoFisher Scientific (USA).

Equipment's

RodVent small-animal ventilator, Opure 10 (10 L portable oxygen cylinder), reptile heating pad (35–40°C), blanket (to cover the animal), Bio-bubble system (Animal Housing and experiment, bio-bubble complete system, 768769), individually ventilated cages (IVC), heating water bath, surgical lamp, autoclave, clear, open field apparatus: a clear Acrylic box (5mm thickness, 610 mm width, and 410 mm height), metal running wheel for pets (32 cm), and stainless-steel grid (40 x 60 cm), automatic tissue processor and automatic H and E (Hematoxylin and eosin) staining apparatus.

Study group's

Eighteen rats ranging between 300-400g weight were randomly assigned to three groups (n = 6/group): (1) sham or healthy (H), in which animals underwent laminectomy surgery without any compression and treatment; (2) UT compression injury animals, in which rats underwent calibrated compression laminectomy with a lesion but without treatment for 28 days; the UT group mean vehicle control, the animal was administered with an equal amount of normal saline solution without the drug and (3) T rats, in which rats underwent calibrated compression laminectomy and received 10 mg/kg/day oral daily dose of MLC901 for 28 days. All calibrated compression-injured rats underwent compression for 15 sec with a similar magnitude of force, which was confirmed by the pilot study, before this study.

2. Methods

Behavioral evaluation before surgery

Rats were subjected to behavioral evaluation before surgery for 7 days, on day 3, and day 7 before surgery. During the pre-injury evaluation and training, animals were evaluated for body weight, physical evaluation, and locomotion functions evaluation i.e. running wheel, grip strength test, and sensory function, to record the baseline values. During the physical examination for 7 days, each animal was assessed on these six categories (body weight, physical appearance, behavior/activity, clinical signs, and bladder functions).

Surgical procedure for compression SCI

The detailed surgical preparation was carried out as previously described in our published protocol (Anjum, Cheah et al. 2022). In brief, rats were anesthetized by intraperitoneal injection of Ketamine/xylazine cocktail at 0.1 mL/100 g of animal body weight before the surgical procedure. The body temperature was maintained at 37 ± 0.5 °C. Before surgery, rats were placed in a supine position and were shaved from the thoracic and lumber region so that the surgical area was marked and cleaned. Under aseptic conditions, a 10 cm midline incision was made, and the spinal cord was exposed. Before compression, tramadol was administered subcutaneously for analgesic activity. After compression injury, the muscles were sutured, and the animals were kept under observation until they gained consciousness and were found active. Both injury groups were compared to the sham group to estimate the magnitude of the injury, and the treated (T) and untreated (UT) groups were compared to evaluate the regenerative potential of MLC901.

Administration of MLC901

The T rats were injected with a daily dose of 10 mg/kg/day in drinking water by optimizing the water update per day. If the animal did not take the required number of water-containing drugs was fed to the animal using oral gavage 26G. The dose of MLC901, frequency of administration, and length of the MLC901 were selected based on the prior publications (Ebrahimi-Ghiri, Alijanpour et al. 2021, Islam, Hatta et al. 2022).

Postoperative Care

After the spinal injury, 0.4 mg/100 g subcutaneous tramadol twice daily for 3–5 days was administered to alleviate pain symptoms. Soft food and autoclaved clean water were provided nearby and easily accessible to the animals. The animal's daily food and water intake were monitored carefully, and if the animal did not feed and drink properly, Ringer lactate solution (1 mL/100 g body weight subcutaneously) was administered 3–5 days post-injury. Urinary retention typically occurs because of lower limb paralysis. To avoid this, the bladder was manually massaged twice daily to facilitate urination: the abdomen was gently palpated to locate the bladder, and then gentle downward pressure was applied until the bladder was empty. In case of bloody urination, the antibiotic Baytril (100 mg/mL) at 50 mg/100 g body weight was injected subcutaneously. The rats' weight was controlled by optimizing the food intake to control unnecessary weight gain in rats. According to guidelines, rats need approximately 60 calories per day. The equation used to determine the amount of food needed is 5 to 6 grams (0.16 to 0.19 oz) of food per 100 grams (3.22 oz) of body weight of the rat per day. Feed consumption would be between 15 and 20 grams per day for an average adult rat.

Quantification of Basso, Beattie, and Bresnahan (BBB) scores

The BBB 21-point open-field locomotor rating scale was used to evaluate hind limb motor function; 0 points indicated total paralysis of the hind limbs, and 21 points indicated full functioning and normal locomotion. Behavioral analyses were conducted at the indicated time points (3 and 7 days before surgery and 3, 7, 14, 21, and 28 days after surgery) by two blinded investigators.

Running Wheel assessment

The running wheel is one of the most widely studied behaviors in laboratory rodents. It is frequently used to evaluate motor deficits in rodent models having brain and spinal cord injuries (Lee, Cao et al. , Engesser-Cesar, Anderson et al. 2005, Engesser-Cesar, Ichiyama et al. 2007). In this test, the animals were placed into the running wheel and then the wheel was rotated forcefully at 90°. The animal tried to restrain the wheel movement by gripping it from the forelimb and hindlimb and the time taken by the animal to restrain the wheel was recorded for both groups, as reported previously (Engesser-Cesar, Ichiyama et al. 2007). The scale used was described below (0-4).

Recovery in grid walking pattern

The grid apparatus consisted of a square metal grid (40 x 60 cm), which was cleaned with 70% ethanol and placed in the open field apparatus. The animals were placed at one edge of the grid and were allowed to walk and reach another edge. The walking behaviors such as the number of faults, total footsteps, and time taken to walk the grid were recorded as described previously and were then analyzed (Pajoohesh-Ganji, Byrnes et al. 2010).

Grip strength test using inverted grid method.

The grip strength test is utilized to estimate muscle strength pre- and post-injury (Stępniewska, Budnik et al. 2019). Usually, rats can easily grip a grid for 30-40 s in an inverted position. Under cognitive and neurological disorders, the animal grip strength was reduced (Stępniewska, Budnik et al. 2019). The grip strength following injury was carried out by placing the rat upside down on a grid approximately 20 cm above the ground, and a timer was set, the maximum amount of time the rat withstands and holds gird in an inverted position was recorded.

Somatosensory Evoked Potentials (SEPs)

SEPs of the animals were recorded 0, 2, and 4 weeks postoperatively, and sensory and motor nerve conduction was detected with a Nicolet® Viking Quest™ apparatus from the United States. After general anesthesia, the sciatic nerve was located targeting the thigh bone, to which stimulating and recording electrodes were connected, respectively. The stimulating electrode was subsequently fixed to the hind legs and recording electrodes were placed on the hindlimb cortical sensory area between the coronal suture and sagittal suture lines. The reference electrodes were placed 0.5 cm posterior to the recording electrodes, eliciting direct-current square wave electrical stimulation with an intensity of 10–30 mA, a pulse width of 0.1 ms, and a frequency of 0.1 Hz was superimposed for 50-60 times repetition. A single-pulse stimulation with a duration of 50 ms was used. The SEPs latency, duration, and amplitude were recorded, and compared with healthy and pre-injury values.

Spinal Cord Extraction

After 4 weeks post-surgery and treatment, the rats were sacrificed with an intraperitoneal injection of pentobarbital (200 mg/mL). The skin at the dorsal of the spinal cord was cut and any excess tissue surrounding the length of the spinal column was cleared. The actual level of the laminectomy and injury can be confirmed by counting ribs again. The Vanna scissors were used to displace the small sections of the spinal column in a caudal-to-rostral direction until the cord was exposed enough to allow safe removal. After removal of spinal tissue, place spinal cord tissue in 4% PFA (paraformaldehyde) and allow the tissue to fix for 24h at 4 °C. The remaining spinal cord tissue was preserved by incubating in 30% sucrose solution for 24h at 4 °C and then placed in a -20°C freezer for long tissue storage.

Histology

The transverse thoracic vertebral block was fixed in 4% paraformaldehyde in PBS overnight at 4°C. The fixed spinal cord blocks were placed in an automatic tissue processor to process the tissue for H and E staining. The processed tissue was placed in a mold with molten paraffin solution and completely solidified by keeping it on dry ice if used immediately or stored at 4°C and cut into 5-μm thick sagittal and parasagittal sections using a microtome. The cut sections were placed on glass slides and dried overnight at 40°C. The dried slides were then placed in an automatic H and E staining machine (Leica autostainer ST5010). The stained slides were covered with a drop of

mounting solution (Paramount) and then covered with a cover slip. The cover slip was pressed down on all sides to ensure the proper distribution of fluid. These slides were allowed to dry overnight and then viewed under a microscope. The Hematoxylin and eosin staining were performed for general histological examination under microscope observation (Olympus, Tokyo, Japan). To quantify the density of neurons in the anterior horn, the total area of neurons per 100 µm2 was measured using Image J software. It was expressed as a percentage relative to that of the sham. Broken sections were excluded from the quantification. We selected six sections/groups, so it will be 1 longitudinal and 1 transverse section/ animal, and have quantified n=3, images from each section of tissue, so we have n=6/ group and did three images/ tissue, so the total will be 18 images/ group.

Immunohistochemical assay

Immunohistochemical staining was performed according to the manufacturer's instructions. Briefly, paraffin-embedded spinal cord tissues were cut into 5-µm sections and deparaffinized with 100 % xylene. Then, antigen retrieval with 100 µL of antigen retrieval solution for 15 minutes at 96 °C was performed, and the sections were incubated with 10 % goat serum at room temperature for 20 minutes. The sections were incubated with rabbit monoclonal antibodies GAP43 (1:200) and mouse GFAP (1:200) at 4°C overnight and then incubated with goat anti-rabbit IgG (488, 1:400) and goat anti-mouse IgG (594, 1:400), respectively, for 30 minutes at 37°C. Then, the sections were incubated with DAPI (1:15000) prepared in DPBS. This solution was applied to tissue (dome) and incubated for 20-40 minutes at ambient temperature in the dark, followed by washing with DPBS 3X (5 minutes each). Positive immunostaining was defined as green and red fluorescence staining. PBS was used instead of a primary antibody in the negative control group. The stained and washed slides were mounted with one drop of mounting solution and observed under the Nikon A1R Confocal Microscope System.

Histological examination of liver and Kidney tissue

The specimens were fixed in 10% formalin for 24 h, and standard dehydration and paraffin-wax embedding procedures were used. Sections (5 µm) were cut in a microtome and adhered to glass slides with polylysine. Hematoxylin and eosin-stained slides were prepared using standard methods and evaluated by light microscopy.

Supplementary data 2. Figures

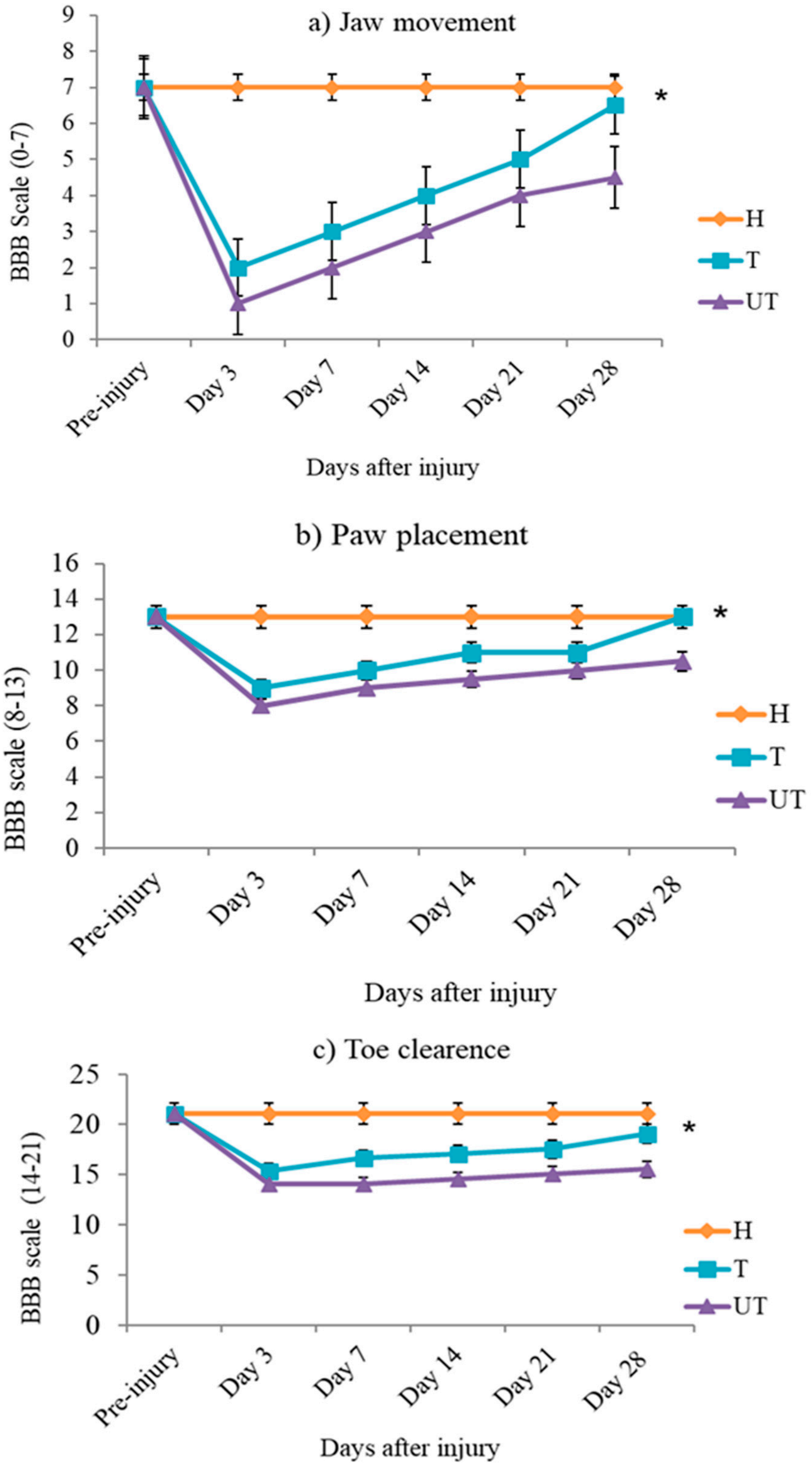

Figure S1: BBB sub score, for i.e. jaw movement (0-7), paw placement (8-13), and toe clearance (14-21) between T and UT rats. The T rats showed significance ($p<0.05$) compared to UT rats, with data presented as mean and SD, n=5.

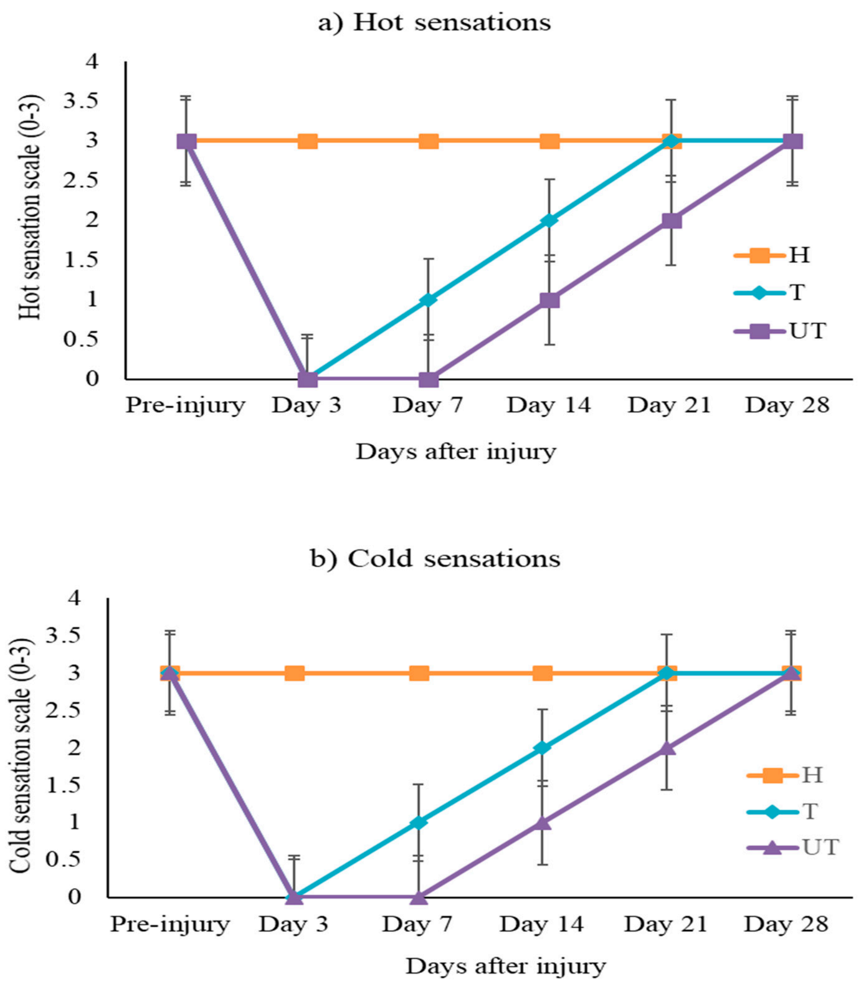

Figure S2: Sensory function assessment for a) hot, and b) cold sensations, indicated the sensory coordination in H, T, and UT rats, the hot and cold sensation was scored on a 0-3 scale, with a 0 score for no response and a 3-score showing quick response. The T rats scored better than the UT rats.

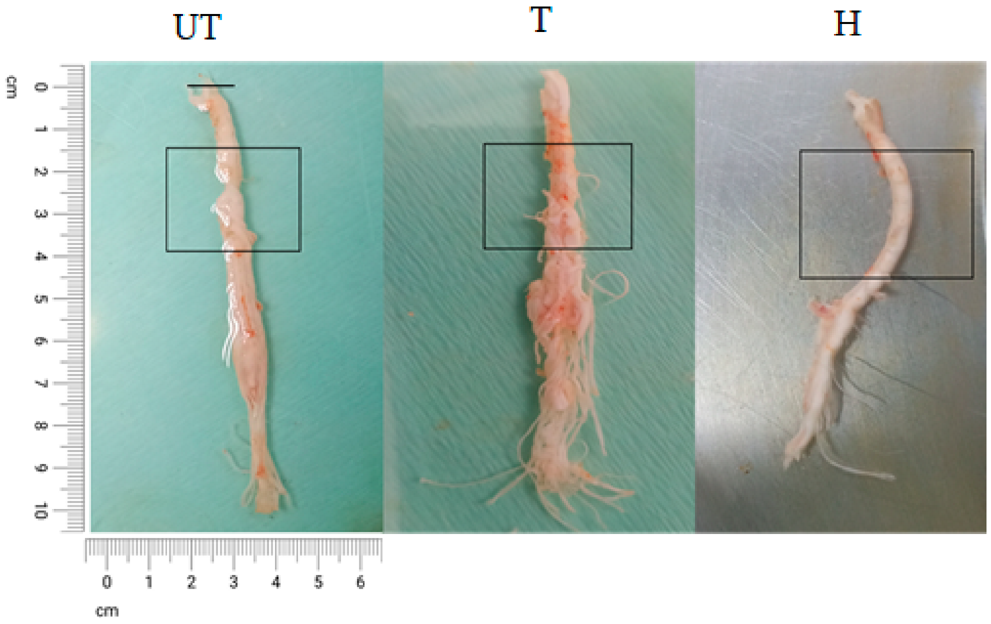

Figure S3: The photograph of the extracted spinal cord from different, UT and T rats, the black box indicated the compression area in UT and T rats compared to H rats.

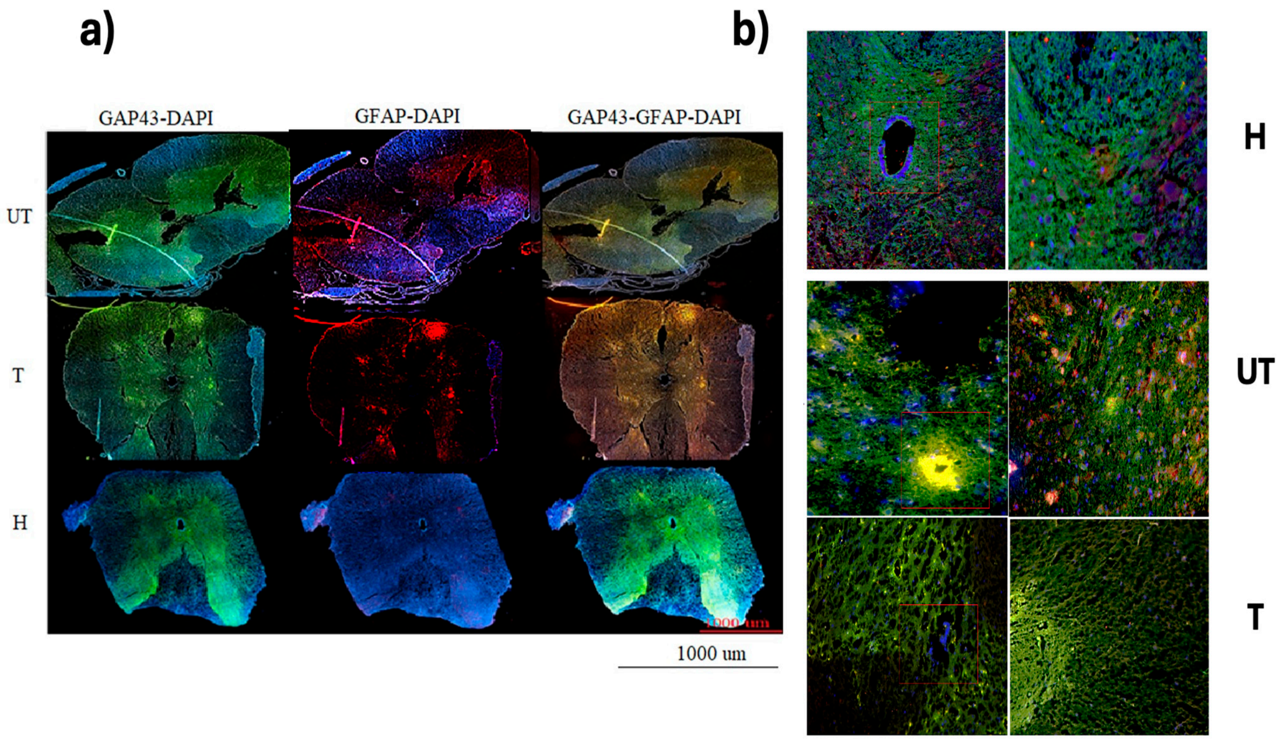

Figure S4: Immunohistochemistry (IHC) analysis showing expression of GAP43 indicating the presence of motor neurons (green fluorescence), GFAP showed astrocytes and glial cells (red fluorescence), and DAPI (blue fluorescence) stain cell nucleus. The lower expression of GAP43 and GFAP was observed in both UT and T injury rat tissue compared to H rat tissue. Images were taken at a scale bar of 1000 µm, with 10X magnification, n=5.

Supplementary Videos: provided in separate files with titles listed below:

Video S1: The calibrated forceps compression injury model.

Video S2: Treated (T) rats after 2 weeks of injury.

Video S3: Untreated (UT) rats after 2 weeks of injury

References

Anjum, A., Y. J. Cheah, M. D. i. Yazid, M. F. Daud, J. Idris, M. H. Ng, A. S. Naicker, O. H. Ismail, R. K. Athi Kumar, G. C. Tan, Y. P. Wong, M. K. Mahadi and Y. Lokanathan (2022). "Protocol paper: kainic acid excitotoxicity-induced spinal cord injury paraplegia in Sprague–Dawley rats." Biological Research **55**(1): 38.

Ebrahimi-Ghiri, M., S. Alijanpour, F. Khakpai and M.-R. Zarrindast (2021). "Antidepressant efficacy of MLC901 in the 6-hydroxydopamine mice model of Parkinson's disease." Physiology and Pharmacology **25**(4): 328-333.

Engesser-Cesar, C., A. J. Anderson, D. M. Basso, V. Edgerton and C. W. Cotman (2005). "Voluntary wheel running improves recovery from a moderate spinal cord injury." Journal of neurotrauma **22**(1): 157-171.

Engesser-Cesar, C., R. M. Ichiyama, A. L. Nefas, M. A. Hill, V. R. Edgerton, C. W. Cotman and A. J. Anderson (2007). "Wheel running following spinal cord injury improves locomotor recovery and stimulates serotonergic fiber growth." European Journal of Neuroscience **25**(7): 1931-1939.

Islam, A. A., M. Hatta, W. Adhimarta, M. Faris, N. Mustamir, A. Bukhari, C. Kaelan, J. Hendarto, N. H. Imran and R. M. Rosyidi (2022). "The role of MLC901 in reducing VEGF as a vascular permeability marker in rats with spinal cord injury." Annals of Medicine and Surgery **75**: 103344.

Lee, D.-H., D. Cao, Y. Moon, C. Chen, N.-K. Liu, X.-M. Xu and W. Wu "Enhancement of motor functional recovery in thoracic spinal cord injury: voluntary wheel running versus forced treadmill exercise." Neural Regeneration Research: 10.4103.

Pajoohesh-Ganji, A., K. R. Byrnes, G. Fatemi and A. I. Faden (2010). "A combined scoring method to assess behavioral recovery after mouse spinal cord injury." Neuroscience research **67**(2): 117-125.

Stępniewska, A., M. Budnik, K. Krzemiński, W. Niewiadomski, A. Gąsiorowska, G. Opolski, J. Kochanowski, K. Mieczkowska, K. Żukowska and K. Szepietowska (2019). "Impaired hemodynamic response to tilt, handgrip and Valsalva manoeuvre in patients with takotsubo syndrome." Autonomic Neuroscience **220**: 102555.
